# PAINscape—Exploring patient experiences with ketamine for chronic neuropathic pain: A qualitative study

**DOI:** 10.1080/24740527.2026.2615473

**Published:** 2026-03-04

**Authors:** Nandana Parakh, Danielle Lessor, Kevin Dang, Paul Ritvo, Duminda N. Wijeysundera, Victoria Tucci, Mariela Leda, Mindy Lu, Gabriella Mattina, Janneth Pazmino-Canizares, Zaaria Thomas, Roshni Nayar, John G. Hanlon, Sérgio M. Pereira, Karim S. Ladha, Hance Clarke, Sakina J. Rizvi, Cheryl Pritlove, Akash Goel

**Affiliations:** a Temerty Faculty of Medicine, University of Toronto, Toronto, ON, Canada; b Department of Anesthesiology, Pharmacology and Therapeutics, University of British Columbia, Vancouver, BC, Canada; c School of Kinesiology and Health Sciences, York University, Toronto, ON, Canada; d Department of Psychology, York University, Toronto, ON, Canada; e Department of Anesthesia, St Michael’s Hospital, Unity Health Toronto, Toronto, ON, Canada; f Department of Anesthesiology and Pain Medicine, University of Toronto, Toronto, ON, Canada; g Department of Anesthesia and Pain Management, Toronto General Hospital, University Health Network, Toronto, Ontario, Canada; h Department of Psychiatry, University of Toronto, Toronto, Ontario, Canada; i ASR Suicide and Depression Studies Program, St. Michael’s Hospital, Toronto, ON, Canada; j Applied Health Research Centre, Li Ka Shing Knowledge Institute, St. Michael’s Hospital, Toronto, ON, Canada; k Social and Behavioural Health Sciences, Dalla Lana School of Public Health, Toronto, ON, Canada

**Keywords:** Ketamine, chronic pain, chronic neuropathic pain, pain reduction, anesthesia

## Abstract

**Background/Aims:**

Chronic pain affects approximately 8 million Canadians annually and is defined by persistent pain lasting over 3 months. Ketamine is an anesthetic drug used to treat chronic neuropathic pain, a subset of chronic pain. To better understand ketamine’s therapeutic benefits and feasibility as a treatment for chronic neuropathic pain, it is important to characterize patient experiences and perspectives with ketamine, as well as barriers and facilitators to accessing this treatment.

**Methods:**

Thirteen participants were recruited from the chronic pain ketamine infusion program at St. Michael’s Hospital in Toronto, Canada. Each participant completed a survey that captured demographic information and chronic pain features, followed by a semistructured interview. Interview data were analyzed, and themes were generated using content analysis.

**Results:**

All participants described decreased pain intensity and increased functionality after receiving ketamine treatment. Barriers to ketamine treatment included fragmented health systems and long wait times, along with a struggle for pain validation by health care providers. Facilitators of ketamine treatment included support from individual health care providers and the provision of a supportive treatment environment.

**Conclusions:**

Although pain experiences differed among participants, all participants reported decreased pain with ketamine infusions. Addressing the stigma associated with ketamine infusions, further research around augmenting durability of ketamine, and providing a safe treatment environment can all improve ketamine’s benefit for chronic neuropathic pain. Understanding the barriers and facilitators, as well as implementing participant suggestions, will not only help inform our ketamine program but can improve access to pain management and facilitate future research in this field.

## RÉSUMÉ

**Contexte/Objectifs :** La douleur chronique affecte environ huit millions de Canadiens chaque année et se définit comme une douleur persistante pendant plus de trois mois. La kétamine est un anesthésique utilisé pour traiter la douleur neuropathique chronique, une forme particulière de douleur chronique. Afin de mieux comprendre les bénéfices thérapeutiques et la faisabilité de la kétamine en tant que traitement de la douleur neuropathique chronique, il est important de caractériser les expériences et les perspectives des patients à l’égard de ce traitement, ainsi que les obstacles et les facteurs facilitants liés à son accessibilité.

**Méthodes :** Treize participants ont été recrutés dans le cadre du programme de perfusion de kétamine pour la douleur chronique de l’hôpital St. Michael’s à Toronto, Canada. Chaque participant a d’abord répondu à un questionnaire visant à recueillir des données démographiques et les caractéristiques de sa douleur chronique, puis a participé à un entretien semi-structuré. Les données issues des entretiens ont fait l’objet d’une analyse de contenu ayant permis de dégager des thèmes.

**Résultats :** Tous les participants ont décrit une diminution de l’intensité de la douleur et une amélioration du fonctionnement après avoir reçu un traitement par kétamine. Les obstacles au traitement par kétamine comprenaient la fragmentation des systèmes de santé et les longs délais d’attente, ainsi que des difficultés liées à la validation de leur douleur par les professionnels de la santé. Les facteurs facilitants du traitement par kétamine comprenaient le soutien de certains professionnels de la santé et la mise en place d’un environnement thérapeutique favorable.

**Conclusions :** Bien que l’expérience de la douleur diffère d’un participant à l’autre, tous ont signalé une diminution de la douleur après les perfusions de kétamine. La réduction de la stigmatisation associée aux perfusions de kétamine, la réalisation d’autres recherches portant sur la prolongation de la durée de leurs effets, ainsi que la mise en place d’un environnement thérapeutique sécuritaire pourraient accroître les bénéfices de la kétamine dans le traitement de la douleur neuropathique chronique. La compréhension des obstacles et des facteurs facilitants, de même que la mise en œuvre des suggestions formulées par les participants, contribueront non seulement à orienter notre programme de kétamine, mais aussi à améliorer l’accès à la prise en charge de la douleur et à soutenir les recherches futures dans ce domaine.

## Introduction

Chronic pain is defined as pain persisting for more than 3 months and affects approximately 8 million Canadians a year.^1^ Chronic neuropathic pain (CNP) is a subset of chronic pain caused by injury to or disease of the somatosensory nervous system.^2,3^ This pain is often characterized by sensations of burning, shooting, stabbing, and electrical shocks, but other sensations can also occur.^2,3^ Chronic pain can have a variety of physical, social, functional, and economic impacts, leading to increased rates of comorbidities such as depression, diabetes, and heart disease, along with poorer quality of life, limited mobility, and decreased physical activity.^4^ The economic burden of chronic pain is significant, with total direct and indirect costs in Canada in 2019 estimated between $38.2 and $40.3 billion.^1^

The urgent need for more effective, feasible, and accessible treatments has increased the interest in ketamine, an anesthetic drug with multiple uses including the treatment of CNP.^5,6^ Ketamine was first approved for in-hospital use in 1970.^7^ It is a noncompetitive antagonist at the phencyclidine binding site of the N-methyl-d-aspartate central nervous system receptors.^4^ Ketamine has been proven to have psychomimetic, antidepressant, and, importantly, analgesic effects.^4,8^ Quantitative research demonstrates evidence of short-term pain relief of CNP with ketamine infusions.^9^ Much of the literature on ketamine has focused on its effects on treatment-resistant depression, with limited qualitative research on its application in treating CNP.^10^ Furthermore, there is a lack of understanding around patient experiences regarding the administration of ketamine for CNP, which could impact the way ketamine is prescribed and utilized for pain.

This qualitative study explored patients’ experiences and perceived impacts of ketamine treatment in their management of CNP. Our ketamine infusion program is funded through the Ontario Health Insurance Plan. There are no out-of-pocket expenses for patients. Our goal was to better understand patient-reported barriers to the treatment of CNP and factors influencing health care availability for this patient population. Additionally, we sought to understand factors that facilitated access to treatments for CNP to inform future directions of patient-centered chronic pain care. Lastly, we wanted to examine various systemic and patient- level determinants of engaging in ketamine treatment, to better understand motivations for trialing ketamine, and factors that may hinder the acceptance of this treatment. By exploring patient perspectives around ketamine, as well as its accessibility and impact, we aim to improve access to treatments for CNP.

## Methods

### Study design

Ethics approval was obtained from the Unity Health Research Ethics Board (REB) prior to beginning the study (Ethics Approval Number REB 20-113). Prior to patient participation in the study, both verbal and written informed consent were obtained.

We adopted a qualitative descriptive study design to explore patient experiences with ketamine infusion therapy for CNP. Qualitative descriptive methods are widely utilized in health care research, particularly in areas where evidence is limited, to delve into lived experiences and to address critical clinical, service, and policy challenges.^11^ This approach acknowledges the subjective and diverse nature of individual experiences, grounding our findings in the real-world challenges and perspectives of participants.

### Participant selection

**Figure 1.**
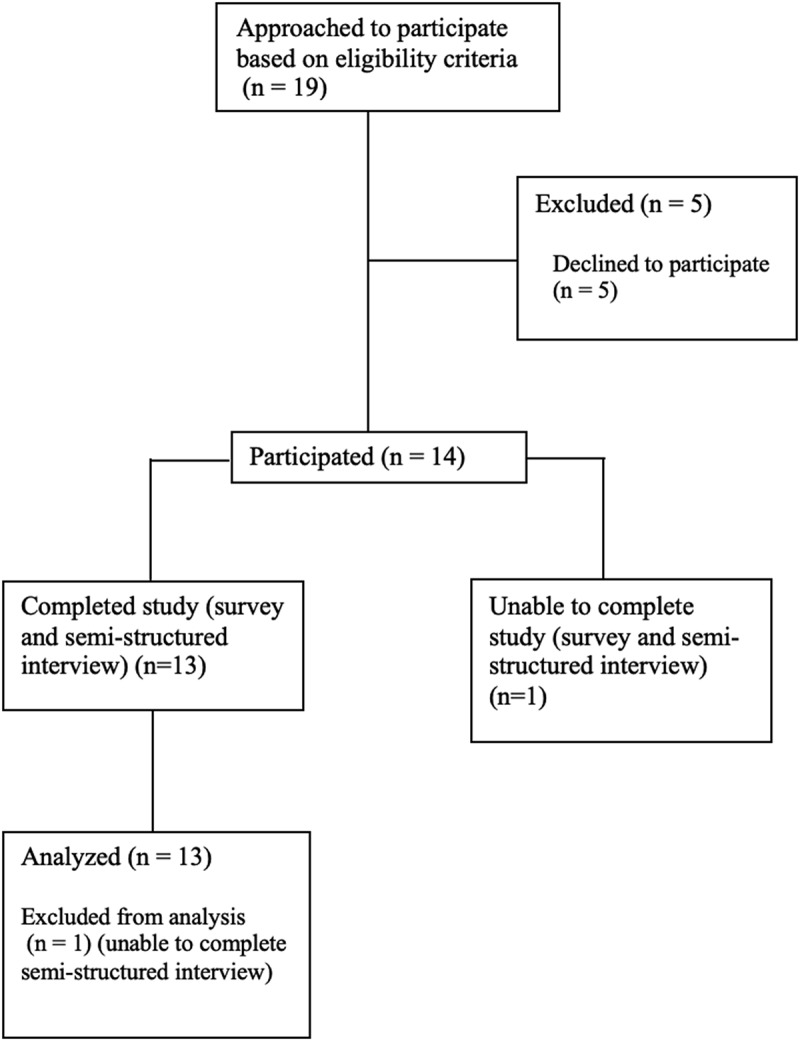
Flow diagram for participant recruitment.

Participants were recruited from the chronic pain clinic at St. Michael’s Hospital in Toronto, Canada. The selected participants included all participants presenting to the clinic for a ketamine infusion for pharmacological pain management between July 23, 2024, and August 13, 2024, on the days the researchers visited. Participants who were 18 years or older, diagnosed with CNP (>3 months), receiving in-person ketamine infusions for pharmacological pain management, and able to complete both the survey and semistructured interview were included in the study. Participants had to have received at least one ketamine infusion to participate in the study. Patients not receiving ketamine infusions and those who did not possess sufficient communication abilities in the English language were excluded. Nineteen participants were approached based on eligibility criteria, and 14 participants elected to participate in the study. One participant was unable to complete the interview and was subsequently excluded from the study. Please see Figure 1 for a flow diagram of participant recruitment.

### Data generation

A data analysis and statistical plan was written and filed with our institutional review board before data were accessed. A survey was administered to collect demographic information and baseline treatment-related factors specific to their CNP. The survey included multiple-choice questions, binary responses (e.g., yes or no), and short-answer questions (Appendix A). Upon completing the survey, participants took part in a semistructured interview designed to explore their experiences with CNP and the barriers and facilitators associated with ketamine infusions (Appendix B). A semistructured interview approach was chosen for its flexible format, which allowed for the collection of open-ended data and a deeper understanding of participants’ perspectives.^12^ All interviews were conducted in English and held in person at the St. Michael’s Hospital chronic pain clinic.

Survey responses and audio recordings were securely stored on a password protected hospital server. Survey responses and interview transcripts were deidentified and labeled with an alphanumeric code.

### Data analysis

The interviews were conducted, audio-recorded, and transcribed by the lead author (N.P.), who also ensured the removal of identifiable information to protect participants’ privacy. Transcripts were uploaded to NVivo 12 software to facilitate systematic data organization and analysis.^13^

Thematic saturation of the data was continually evaluated. Based on the protocol, we targeted a minimum sample size of 12 participants. Once reaching 12 participants, transcripts were analyzed, at which point it was determined that thematic saturation had been reached. We completed one more interview to ensure full thematic saturation.

Employing content analysis, N.P. and A.G. engaged in multiple readings of the transcripts to ensure a comprehensive understanding of the data.^14^ N.P. developed an initial coding framework by analyzing a subset of four transcripts, which guided the coding process, through an inductive framework. This framework was iteratively refined as additional transcripts were analyzed, resulting in the creation of a detailed codebook containing code names, definitions, and example data. To ensure consistency and reliability, a second round of coding was conducted across all transcripts. Once data coding was completed, major themes were identified and reviewed in relation to coded extracts to ensure they reflected participants’ voices.

## Results

### Survey results

A total of 13 individuals participated in the study, at which point it was determined that thematic saturation had been reached.^15,16^ Participants ranged in age from 23 to 74. The sample for this study was notably well-educated, with the majority of participants having attained higher levels of formal education. Nearly half of participants completed graduate school (46%), and an additional five completed postsecondary education (38%), collectively accounting for 84% of the sample. Full participant characteristics are reported in Table 1.

**Table 1.** Participant demographic characteristics.

Characteristic	Participants (*n* = 13)
**Age**	
18–30	4
31–60	5
61–80	4
**Assigned sex at birth**	
Male	3
Female	10
**Gender** (open-text response)	
Woman	3
Female	6
Male	3
Did not answer	1
**Ethnicity**	
European	7
First Nations, Inuit, and Métis	1
Middle Eastern	1
Other	3
Prefer not to answer	1
**Educational attainment**	
Grade/high school (1–12)	2
Postsecondary (13–16)	5
Graduate school (17–20+)	6
**Employment status**	
Employed	4
Retired	4
Student	2
Unable to work	2
Did not answer	1

With respect to chronic pain characteristics, eight participants reported experiencing chronic pain for 1 to 10 years (62%), two for 11 to 20 years (15%), and three for 21 to 30 years (23%). The number of ketamine infusions varied from 1 to 40 infusions over their lifetime. Participants also reported other medications they use for chronic pain, such as selective serotonin reuptake inhibitors (SSRIs), serotonin–norepinephrine reuptake inhibitors (SNRIs), tricyclic antidepressants (TCAs), opioids, and gabapentinoids. Full chronic pain characteristics are reported in Table 2.

**Table 2.** Chronic pain characteristics.

Characteristic	Participants (*n* = 13)
**Length of chronic pain diagnosis**	
1–10 years	8
11–20 years	2
21–30 years	3
**Pain severity**	
0–3	1
4–7	8
8–10	4
**Little interest or pleasure in doing things over the last 2 weeks**	
Not at all	3
Several days	6
More than half the days	3
Nearly every day	1
**Feeling down, depressed, or hopeless in the last 2 weeks**	
Not at all	5
Several days	6
More than half the days	1
Nearly every day	1
**Number of ketamine infusions**	
1–10	5
11–20	3
21–30	2
31–40	2
Multiple	1
**Other medications for chronic pain**	
SSRIs (i.e., sertraline, paroxetine, fluoxetine)	7
SNRIs (i.e., venlafaxine, duloxetine)	6
TCAs (i.e., amitriptyline, imipramine, nortriptyline)	5
Opioids (i.e., morphine, oxycodone, codeine, etc.)	8
Gabapentinoid (i.e., gabapentin, pregabalin)	7
Other	5
**Other treatments for chronic pain**	
Yes	6
No	7
**Treatments for depression–Past**	
Yes	6
No	7
**Treatments for depression–Present**	
Yes	5
No	8

### Semistructured interview results

We identified three overarching themes from the semistructured interviews: (1) impact of ketamine on pain, (2) barriers to accessing pain relief, and (3) facilitators to chronic pain treatment. A brief overview of each theme along with sample illustrative quotes is provided below, followed by a more detailed reporting of results and quotes in Appendix C.

Though the degree and consistency of pain relief varied among participants and across individual infusion experiences, all participants reported that ketamine significantly reduced pain and improved their quality of life (100%). Ketamine had not only a physical impact but a profound mental and emotional impact on participants. Most participants described their CNP as profound and life-altering, with some expressing that its intensity left them yearning for an escape, even through death, to find relief. Ketamine infusions helped reduce the pervasive fear associated with CNP and helped to restore a sense of self and agency. Many participants also noted that ketamine infusions allowed a return to daily activities and overall contributed to improved quality of life. Table 3 provides sample illustrative quotes.

**Table 3.** Sample illustrative quotes around impact of ketamine on pain.

Participant ID	Participant quotes
PID 002	“Well probably the most significant thing with the ketamine infusions that I was living with soul-destroying pain. It was ruining my life to the point that I didn’t want to go on living. The ketamine has resolved the pain issue, it’s amazing.”
PID 003	“I’ll feel no pain when I walk out of here. So, there’s an immediate relief in that sense. But the gradual relief, my opinion of ketamine, is more from the mental side. You’re positively thinking about the outlook of your recovery, the trajectory of your recovery.”
PID 001	“I always just look forward to being able to feel like me again afterwards and being able to do things a little bit more than I can do the last few weeks, and getting back into that.”

Despite the profound relief and renewed functionality that ketamine infusions provided, participants also revealed substantial barriers to accessing this treatment and other essential pain management supports, including, fragmented systems and wait times, infusion interval and inconsistencies, and the struggle for pain validation, which encompasses both a general invalidation of chronic pain, along with a lack of acceptance of ketamine infusions as a viable treatment for CNP. Participants described the arduous task of navigating between multiple clinics and providers, with each step contributing to frustration and delays in receiving essential treatment. Furthermore, many participants stated that infusion intervals had increased due to budget constraints and resource availability (54%), leading to increased pain and decreased quality of life. Lastly, 54% of participants reported that their pain was initially dismissed or undiagnosed. The intangible and often invisible nature of chronic pain, where individuals may appear outwardly “normal” or lack clear indicators of suffering, frequently leads to skepticism or dismissal from health care providers, rendering it difficult to secure needed care. Participants also experienced stigma around utilizing ketamine for pain management. Table 4 provides sample illustrative quotes.

**Table 4.** Sample illustrative quotes around barriers.

Participant ID	Participant quotes
PID 004	“The wait times can be really bad. I think I’ve been to seven different pain clinics for different purposes and each time, its 6 months to a year, if not a year and a half or 2 years, so that’s the biggest [barrier].”
PID 010	“[Ketamine] lasts around 3-ish weeks, and I would get it every 4 weeks. But now it has to be every 5 to 6 weeks, and that’s sad. I now have to use my wheelchair a lot more, because I’m in too much pain to walk, for the last weeks of it, since it’s been longer.”
PID 005	“A lot of people, including my pain specialist, did not believe me. That’s the truth.”

Though there were significant barriers to accessing ketamine infusions, participants also spoke about factors that improved their overall experience with ketamine infusions, such as comfortable and supportive environment, support from individual health care providers, and geographical factors. A secure and comfortable environment for participants led to increased feelings of safety and improved the overall treatment experience. Access to health care providers who were inclusive and believed patients about their pain were able to facilitate the entire treatment process. Physical proximity to the infusion centers was also a significant facilitator. Table 5 provides sample illustrative quotes.

**Table 5.** Sample illustrative quotes around facilitators.

Participant ID	Participant quotes
PID 002	“I feel safe, is the other thing. I think safety, feeling safe, is very important.”
PID 004	“Having a family doctor that acknowledges the chronic pain helps.”
PID 012	“I live [close], so I’m very close to all the hospitals and all the doctors’ offices, which makes things very easy for me, and am able to take cabs places, so makes things more accessible.”

Additional participant quotes and full semistructured interview results are included in Appendix C.

## Discussion

This study explores the experiences of 13 patients receiving ketamine infusions for CNP to better understand the impact of treatment and the barriers and facilitators to treatment access. Our findings can be broken up into three major themes: (1) impact of ketamine on pain, (2) barriers to accessing pain relief, and (3) facilitators to chronic pain treatment.

### Positive impact of ketamine on pain

All participants found that ketamine infusions improved both physical and emotional pain, and many found that it transformed their lives. Even though the extent and duration of relief varied, many explained that ketamine helped to transform their lives by alleviating not only the physical pain but also its emotional toll (e.g., the fear of pain). Notably, ketamine helped to restore a sense of self and agency, along with improving participants’ functionality and quality of life. Indeed, even short periods of relief from pain were described as impactful, restoring functionality and enabling a return to daily activities. These findings are concordant with other studies that found that, overall, ketamine leads to clinically significant reductions in chronic pain, which in turn leads to improved functionality and quality of life.^4,5,9,17,18^

### Barriers

Participants faced three main barriers. They described how fragmented systems and long wait times produced frustration and delays in achieving pain relief. These problems could be addressed by streamlining pain management systems. One approach to streamlining care involves a multidisciplinary approach to chronic pain management, which incorporates the expertise of both physicians and nonphysicians, along with nonmedical specialists. This approach leads to increased continuity of care, greater psychological advantages, increased pain relief, and improved physical functionality.^19,20^

Another key barrier that was brought up by participants was related to the time interval between ketamine infusions and how it impacted pain relief. Participants often found that the unpredictable intervals between infusions led to pain intensity being perceived as higher because it impacted their expectations of their pain management. Additionally, longer intervals between ketamine infusions due to budget constraints or appointment/resource availability led to poorer pain control. The ketamine infusion rate is patient specific and variable; ketamine treatment programs approach this variability differently. For instance, at the Cleveland Clinic (Ohio), ketamine infusions are given at a rate of 0.5 mg/kg over 40 min, over 5 days. Individuals attend these infusions every 3 to 4 months.^21,22^ Our ketamine infusion program, on the other hand, uses an infusion rate between 0.5 and 1 mg/kg per hour, and the number of hours for the infusion is determined by patient characteristics. Due to the variability in patient preferences regarding how often ketamine infusions are required, the “ideal” interval is patient specific, and further research in this area can help to better explore this. Furthermore, better understanding how to augment the durability of ketamine would be helpful. Other studies are also investigating ketamine-assisted psychotherapy to increase durability and efficacy for chronic pain.^23^ The qualitative work of our study can inform future clinical trials seeking to create more robust and efficacious treatment regimens for chronic pain, because it provides a better understanding of the impact ketamine can have on CNP.

Lastly, participants reported that because pain is complex, subjective, and often stigmatized, they were not always believed about the nature and intensity of their chronic pain in general. Some participants described experiencing pain invalidation and stigma from their health care providers earlier in their care, prior to being referred to specialized pain care. These experiences often delayed referral to pain specialists. Additionally, it often took months and visits to multiple providers to receive an official diagnosis of chronic pain, which led to a more intense perception of pain and negatively impacted pain management. Furthermore, ketamine for CNP is often stigmatized due to its potential for misuse.^24^ Participants reported how popular culture has increased stigma around ketamine and how this led to participants feeling uncomfortable sharing their experiences with ketamine infusions with family members or friends. There is limited research around the stigma associated with ketamine infusions as a treatment for CNP; in contrast, this stigma is better discussed in other domains of ketamine use, such as for treatment-resistant depression.^25,26^ Some authors suggest the need to conduct more research on ketamine to fully elucidate its mechanism of action and address concerns around ketamine’s potential for misuse.^27,28^ Other studies recommend better public education around the use of ketamine for medical conditions, as well as disseminating research around the long-term effects of ketamine.^28^ There is stigma related to ketamine infusions both at the individual patient level and at the health care provider level. It is important to address providers’ beliefs and bias around using ketamine therapeutically.^27,28^ Continued education for health care professionals, along with increased research conducted on the long-term effects of ketamine, can help reduce provider stigma.^29,30^

### Facilitators

Participants reported three main facilitators to receiving ketamine for chronic pain. Having a supportive environment in which ketamine was administered was a significant facilitator, because it increased psychological safety. Our approach is to provide a private treatment area where participants receive ketamine infusions with nursing care before, during, and after their infusion. This improves both physical and psychological safety and helps to improve the overall treatment experience. Research asserts that comfort and care in the treatment setting are important institutional factors that improve the overall patient experience.^31,32^ Widely implementing this model of comfort and care during ketamine infusions can have a positive impact on those living with chronic pain.

Furthermore, individual health care providers can have significant impacts on the overall health of a patient; this has been well established in pain management strategies. Provider and patient alignment in terms of their approach to and expectations of pain management can improve patient outcomes.^27,33^ Therefore, enhancing patient–provider communication, in addition to resolving physician bias (which was reported as a barrier above), is paramount in effectively treating CNP.

Lastly, living near ketamine provision centers was a significant facilitator for treatment access, as stated by various participants. Individuals living farther from urban centers, where ketamine infusions for CNP are typically available, have difficulty accessing care, poorer pain control, and increased perception of pain.^34^ It is well established that rural and remote populations experience higher rates of chronic pain, likely reflecting limited access to specialized chronic pain care.^35–37^ Some suggested strategies to address these disparities in care include interventions like mobile ketamine clinics. Currently, mobile ketamine clinics in the United States offer door-to-door treatments for chronic pain and various mental health conditions for those living in remote areas or those with limited mobility.^38^ Further research on this type of treatment can be considered in Canada, in terms of both feasibility and funding. Furthermore, though the current landscape of ketamine treatment requires patients to be physically present, other parts of the process, such as intake and initial appointments, can be streamlined through virtual care.

In addition to the three major themes, we noted the unusually high level of education among the interviewed participants. This may not reflect the broader representation of participants in our clinic because our selected sample was a convenience sample. We approached 19 participants and 5 declined to participate, which limits our ability to compare the education levels of respondents with nonrespondents. Several factors may contribute to why our sample included participants with a high degree of education. Individuals with more education may be better equipped to overcome the various barriers to initiate ketamine infusion treatment. Furthermore, though the treatment is publicly funded at our site through the Ontario Health Insurance Plan, socioeconomic factors may still favor those with more resources, highlighting a need to improve accessibility and reduce structural barriers to access this treatment.

### Strengths and limitations

In terms of strengths, this study promotes patient-centered care by qualitatively exploring patient perspectives regarding experiences with chronic pain and ketamine treatment to gain an appreciation for barriers and facilitators to treatment. Though similar qualitative studies have been conducted to examine the impact of ketamine on depression, there is limited qualitative research surrounding the impact of ketamine on CNP, making this study an important contribution to pain medicine scholarship.

Our study is limited in its generalizability by its single-site nature, small number of participants, higher than average education level, and uneven gender ratio. Due to these limitations, we were unable to engage in comparative analysis by demographic variables including social determinants of health. Given what is known about the differences in pain experiences and management between men and women, future research exploring the use of ketamine as a pain management strategy should engage in comparative analysis across demographically diverse participants.

### Practical implications

This research has multiple implications for future programmatic development, at both our site and others offering or intending to offer treatment for CNP. Future clinical trials are seeking to create more durable and effective treatment regimens for chronic pain.^23^ Our research can help provide a window into the lived experiences of individuals undergoing ketamine treatment for chronic pain, which can help guide treatment decisions and create more effective programs for managing CNP.

#### Improving communication strategies between health care providers and patients

Many patients with CNP often feel like their pain is dismissed or invalidated by health care professionals. Strategies to improve communication between health care professionals and those living with chronic pain include providing validation and endorsing belief of the patient, active listening and practicing empathy, being open and nonjudgmental, and providing support while collaborating with the patient.^39^ Henry and Matthias proposed a model that incorporates clinician, interaction, and patient factors into communication and encourages setting visit outcomes that both the clinician and patient can work toward.^40^ It is hoped that implementing these strategies into communicating with our patients at every step of the treatment process will allow patients to feel better supported by health care providers and create a sense of belonging, thus fostering healing.

#### Ideal ketamine program

**Figure 2.**
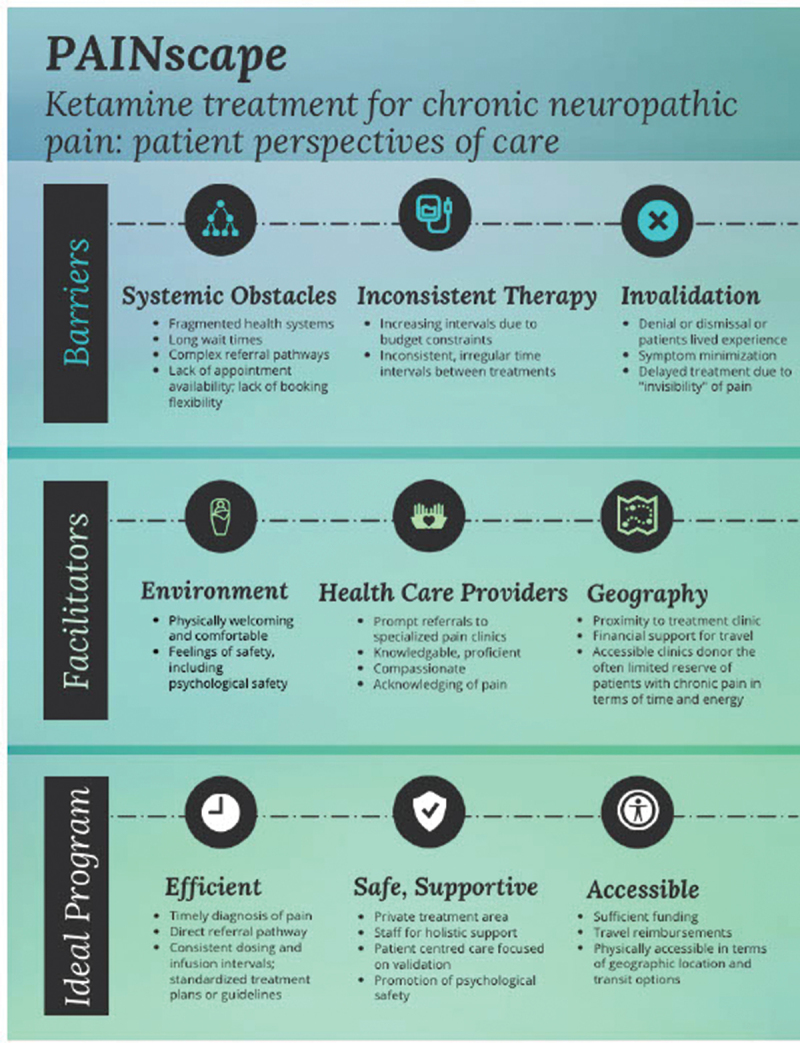
An ideal ketamine infusion program: patient preferences.

An ideal ketamine treatment program should consider patient, clinician, and institutional factors. It is important to note that it is difficult to determine an ideal ketamine infusion interval. Rather, it appears that the ideal interval is patient specific and must be addressed on an individual basis. Figure 2 outlines the general principles that can contribute to creating an efficient, safe, and accessible program for ketamine infusions.

## Conclusion

Chronic pain imposes a significant burden on both individuals living with pain and the health care system. Ketamine has a positive analgesic effect and can significantly relieve pain in those living with CNP. Barriers to ketamine use include long wait times resulting from fragmented health systems, inconsistencies in infusion intervals, accessibility of appointments, difficulty receiving a timely chronic pain diagnosis, and difficulties being believed about chronic pain. Facilitators to ketamine use include a safe and positive treatment environment, support from individual health care providers, and proximity to chronic pain centers. Addressing stigma around CNP and ketamine, along with identifying strategies to provide ketamine to those living at a distance from chronic pain centers, is important in improving the overall ketamine treatment experience. More research is needed to further explore the timing around infusion schedules, along with novel methods for improving ketamine’s durability in treating chronic pain. Overall, these findings will help inform our ongoing work at St. Michael’s Hospital and other pain centers in providing ketamine to improve pain management and quality of life for those with CNP.

## Supplementary Material

PAINscape_CJP_Jan 2 2026_Tracked Changes.docx

## Appendix A: Survey

1. What is your assigned sex at birth?

(a) Male
(b) Female
(c) Intersex
(d) Prefer not to answer

2. Gender. How do you identify? ______________________

3. What is your age? ______________________

4. Ethnicity

(a) African
(b) European
(c) East Asian
(d) South Asian
(e) Southeast Asian
(f) First Nations, Inuit, and Métis (please specify): ____________________
(g) Hispanic or Latinx
(h) Middle Eastern
(i) Other (please specify): ____________________
(j) Prefer not to answer

5. How many years of school have you completed? Please circle one number. If currently enrolled, please indicate highest degree/diploma received.

**Table:**

1 2 3 4 5 6 7 8	9 10 11 12	13 14 15 16	17 18 19 20+
Grade School	High School	Post-Secondary	Graduate School

6. Are you currently … ?

(a) Employed
(b) Self-employed
(c) Out of work and looking for work
(d) Out of work but not currently looking for work
(e) Retired
(f) Student
(g) Unable to work
(h) Prefer not to say

7. How long have you had your diagnosis of chronic neuropathic pain for?

_________________________________________________________

8. What number best describes your pain, on average?^14^

**Table:**

0 (No pain)	1	2	3	4	5	6	7	8	9	10 (Pain as bad as you can imagine)

9. Please answer the following two questions^15,14^:

**Table:**

Over the last 2 weeks, how often have you been bothered by any of the following problems?	Not at all	Several days	More than half the days	Nearly every day
Little interest or pleasure in doing things	0	1	2	3
Feeling down, depressed, or hopeless	0	1	2	3

10. How many ketamine infusions have you had? ______________________________

11. Have you used other medications for chronic pain in the past or are you currently using any medications for chronic pain? Circle all that apply:

(a) SSRIs (i.e., sertraline, paroxetine, fluoxetine)
(b) SNRIs (i.e., venlafaxine, duloxetine)
(c) TCAs (i.e., amitriptyline, imipramine, nortriptyline)
(d) Opioids (i.e., morphine, oxycodone, codeine, etc.)
(e) Gabapentinoid (i.e., gabapentin, pregabalin)
(f) Other (please describe): _____________________________________________

12. Are you currently using other treatments for chronic pain?□Yes □No
13. Have you used treatments for depression in the past?□Yes □No
14. Are you currently using any treatments for depression?□Yes □No

## Appendix B: Semistructured Interview

Participant ID#:_______________________ Date: ___________________________

Location: ____________________________ Researcher:_____________________

Start time____________ End time__________Date of informed consent ____________

Thank you for agreeing to be a part of this study. Today, I will be asking some in-depth questions about your chronic pain and your experiences with using ketamine infusions for chronic pain. The interview will take 30 to 45 min. Please answer the questions to the best of your ability. If you have any questions at any point, please feel free to ask me for any clarifications. All information that you share with me will be kept confidential. If at any point you would like to stop the interview, please let me know. Do you have any questions before we begin the interview?

Since we do not want to identify your responses as belonging to you, when I start the audio recording, please do not provide your name or any other piece of information that identifies you in any of your responses.

**Warm-Up:**

1. What was the infusion experience like, generally?

**Nature of Pain/Experiences with Ketamine:**

(2) During the ketamine infusion experience, did your pain become less or more intense?
  (a) Did your pain experience change? For example, was it more or less central in your experience overall?

(3) Even if pain sensations were not affected, difficult emotions (or thoughts) associated with pain might have changed. Did difficult emotions (or thoughts) change in any way and at any time during your ketamine experience?
  (a) If meaningful changes occurred, even if temporary, can you describe them?
  (b) Were particular thoughts and emotions recurrent or especially memorable?

(4) Can you describe the most unusual experiences during the infusion (sounds, images, memories)? *These could be experiences not currently understood.*
  (a) Were some experiences encouraging or inspiring?
  (b) How might such experiences influence you moving forward?

(5) In everyday life, some activities may make your pain better or worse. Did any of these activities come to mind during your infusion?
  (a) If yes, how did you experience or relate to them?
  (b) Was there a difference from how you usually feel about them?

(6) Were there thoughts or experiences during the infusion that might result in new plans or activities for relieving and reducing pain?
(7) Were there any experiences during the infusion that might influence how you go about enjoying life?
(8) Were there any experiences during the infusion that might influence how you plan and do work?
(9) During the infusion, did you experience changes in how you feel or think about people or relationships in your life?
(10) Have you experienced any barriers to accessing services for chronic pain (i.e., physical, pharmacological, psychological)?
  - Financial barriers?
  - Geographical barriers?
  - Pharmacologic barriers?
  - Cultural barriers?
  - Psychological barriers?
  - Other?
(11) Have you experienced any facilitating factors to accessing services for chronic (some experiences that made it easier to get started with and/or receive treatment)?
  - Financial facilitators?
  - Geographical facilitators?
  - Pharmacologic facilitators?
  - Cultural facilitators?
  - Psychological facilitators?
  - Other?

(12) What were your expectations going into the infusion? Did your experience meet expectations?

### Wrap-Up Question:

(13) Is there anything else you’d like to share about your ketamine or treatment experience, generally, that we didn’t cover?

## Appendix C: Qualitative Results Table

**Table:**

Theme		Analysis	Participant quotes
Impact of ketamine on pain		Ketamine had significant pain reduction impacts, with 100% of participants noting that ketamine reduced their pain. Some also noted cumulative effects of ketamine on their pain, with each subsequent ketamine infusion resulting in more pain relief and decreased total pain. This participant highlights ketamine’s significant impact not only on alleviating physical pain but also on reducing the pervasive fear associated with chronic pain. Most participants described their chronic pain as profound and life-altering, with some expressing that its intensity left them yearning for an escape, even through death, to find relief. For many of the participants, ketamine infusions not only alleviated pain but also restored a sense of self and agency, significantly improving their functionality and quality of life. As expressed by the participant, the ability to “feel like me again” reflects the profound psychological impact of reduced pain, enabling participants to reconnect with their prepain identity and resume activities that chronic pain had previously limited. For these participants, ketamine therapy not only helped to mitigate the physical burden of pain but also fostered a return to normalcy, empowering individuals to engage more fully with their lives.	“My life is a lot better now since I’ve been on the ketamine, because I’m not living in fear of pain all the time.” (PID 002) “Well, probably the most significant thing with the ketamine infusions that I was living with soul-destroying pain. It was ruining my life to the point that I didn’t want to go on living. The ketamine has resolved the pain issue, it’s amazing.” (PID 002) “I’ll feel no pain when I walk out of here. So, there’s an immediate relief in that sense. But the gradual relief, my opinion of ketamine, is more from the mental side. You’re positively thinking about the outlook of your recovery, the trajectory of your recovery.” (PID 003) “I always just look forward to being able to feel like me again afterwards and being able to do things a little bit more than I can do the last few weeks and getting back into that.” (PID 001)
Barriers	Fragmented systems and wait times	Fifty-four percent of participants reported fragmented health care systems and prolonged wait times as significant barriers to accessing chronic pain treatments, including ketamine, which ultimately impeded their ability to receive adequate pain management. For many, accessing a chronic pain clinic was not a straightforward process but rather a protracted journey through a complex and often fragmented health care system. Participants described the arduous task of navigating between multiple clinics and providers, with each step contributing to frustration and delays in receiving essential treatment. Participants faced repeated and lengthy wait times while seeking care, prolonging their journey to receive adequate supports. Such experiences illustrate the systemic hurdles faced by some individuals living with chronic pain, where navigating fragmented referral pathways not only delayed relief but also compounded the psychological and physical challenges of managing their condition.	“It took me a long time. I had to go through so many different doctors to get referred to a pain clinic and find the treatment that I needed, so it was really hard. In terms of accessing treatments, I don’t know that there was anything positive.” (PID 001) “The wait times can be really bad. I think I’ve been to seven different pain clinics for different purposes and each time, its 6 months to a year, if not a year and a half or 2 years, so that’s the biggest [barrier].” (PID 004)
	Infusion interval and inconsistencies	Increased time intervals between infusions were experienced by many participants, most often due to funding constraints and resource availability. These increased time intervals led to increased pain and decreased quality of life, and some participants found that by increasing the time interval between infusions, they noticed a negative functional impact. Participants also noted that knowing that wait times between infusions were increasing due to limited availability, along with the possibility of losing access to treatments due to funding, had a negative impact on their mental health.	“I’ve been coming, I think, every 4 weeks. And I think right now they’re finding that there’s not enough bed spaces to administer the ketamine, so now it’s every 5 weeks. Which is unfortunate because sometimes that last week is really tough. It really should be every 4 weeks for it to be efficacious.” (PID 002) “[Ketamine] lasts around 3-ish weeks, and I would get it every 4 weeks. But now it has to be every 5 to 6 weeks, and that’s sad. I now have to use my wheelchair a lot more, because I’m in too much pain to walk, for the last weeks of it, since it’s been longer.” (PID 010) “I mean, it’s hard to be in pain every day of your life. So to have something like ketamine that works for at least 3 weeks is really great. But now because of the funding and too long wait lists, it has to be longer than that. And then eventually it stops, so that’s kind of hard to conceptualize in my brain, because it’s something that helps but now, eventually, I can’t have it, so am I going to be in more pain for the rest of my life?” (PID 010)
	*The struggle for pain validation*	54% of participants shared that their pain was initially dismissed or went undiagnosed. As illustrated by the participant, perceptions of illness are often rooted in visible or measurable symptoms. Since chronic pain often does not have physical manifestations, individuals often found that they were met with dismissal from health care providers, which was a significant barrier and accessing health care. Some participants also shared that being told their pain was imaginary or “all in their head” not only invalidated their pain but also cast a judgment on their credibility, leading to psychological distress. Lastly, some participants also experienced stigma around ketamine as a pain management strategy from their support networks. Overall, this invalidation of pain management meant that participants were not always given access to pain management strategies, such as ketamine infusions, as readily. This increased overall pain and led to worse health outcomes.	“Because it’s not necessarily something you can see, all the time. For me, I look pretty normal. Pain is invisible, so it’s tough. And I think if you don’t fit a certain [image] of what people expect pain to look like, even your providers, it can impact the care you get.” (PID 008) “A lot of people, including my pain specialist, did not believe me. That’s the truth.” (PID 005) “I mean, you get told a lot that your pain’s all in your head, it’s not real or whatever, and that’s hard to hear because, yeah, no one can see it physically, but to say that it’s just all in your head, you’re making it up for attention, that’s hard to deal with, psychologically, I guess.” (PID 010) “So I don’t tell people, other people who are not a part of my life, what I’m on, or even people in my life, what I’m on, because of the stigma of that drug. It’s horrible.” (PID 005)
Facilitators	Comfortable and supportive environment	Participants noted that the environment they were in during the infusion had a significant impact on their treatment experience. A physically comfortable and private environment with friendly and caring staff led to participants feeling safe during their infusions, which made them more receptive to the treatment and more comfortable overall.	“And everyone that runs the infusions are really amazing, and I think they do a really good job at making it comfortable and listening if you need anything. So it’s just things like that, where they recognize you as a person, rather than just like a patient, and what you’re going do to the patient, it’s more like a humanistic experience.” (PID 010) “I feel safe, is the other thing. I think safety, feeling safe, is very important.” (PID 002)
	Support from individual health care providers	The impact that individual health care providers can have on a participant should not be disregarded. Individual health care providers can facilitate the entire treatment journey, and having a provider who is knowledgeable and validates their patients’ experiences can make the process more efficient.	“Having a family doctor that acknowledges the chronic pain helps.” (PID 004) “My doctor is a really good doctor, I’m so happy to have him. He helped me so much. He’s doing everything he can to help me with this pain.” (PID 014)
	Geographical factors	Lastly, some participants noted that living close to a center where they could receive chronic pain treatment was helpful, because it lessened the burden of transit, saved time, and was more financially feasible.	“I live [close], so I’m very close to all the hospitals and all the doctors’ offices, which makes things very easy for me, and am able to take cabs places, so makes things more accessible.” (PID 012)

## References

[1] Government of Canada. Canadian pain task force report: an action plan for pain in Canada. Ottawa; 2021 May [accessed 2024 Sept 15]. https://www.canada.ca/content/dam/hc-sc/documents/corporate/about-health-canada/public-engagement/external-advisory-bodies/canadian-pain-task-force/report-2021-rapport/report-rapport-2021-eng.pdf

[2] Scholz J, Finnerup NB, Attal N, Aziz Q, Baron R, Bennett MI, Benoliel R, Cohen M, Cruccu G, Davis K, et al. The IASP classification of chronic pain for ICD-11: chronic neuropathic pain. Pain. 2019;160(1):53–19. doi:10.1097/j.pain.0000000000001365. 30586071 PMC6310153

[3] Johannes CB, Le TK, Zhou X, Johnston JA, Dworkin RH. The prevalence of chronic pain in United States adults: sesults of an internet-based survey. J Pain. 2010;11(11):1230–39. doi:10.1016/j.jpain.2010.07.002. 20797916

[4] Cohen SP, Bhatia A, Buvanendran A, Schwenk ES, Wasan AD, Hurley RW, Viscusi ER, Narouze S, Davis FN, Ritchie EC, et al. Consensus guidelines on the use of intravenous ketamine infusions for chronic pain from the American Society of Regional Anesthesia and Pain Medicine, the American Academy of Pain Medicine, and the American Society of Anesthesiologists. Reg Anesth Pain Med. 2018;43(5):521–46. doi:10.1097/aap.0000000000000808. 29870458 PMC6023575

[5] Niesters M, Martini C, Dahan A. Ketamine for chronic pain: risks and benefits. Br J Clin Pharmacol. 2014;77(2):357–67. doi:10.1111/bcp.12094. 23432384 PMC4014022

[6] Buhrman M, Syk M, Burvall O, Hartig T, Gordh T, Andersson G. Individualized guided internet-delivered cognitive-behavior therapy for chronic pain patients with comorbid depression and anxiety: a randomized controlled trial. Clin J Pain. 2015;31(6):504–16. doi:10.1097/AJP.0000000000000176. 25380222

[7] Krupitsky E, Kolp E. Ketamine psychedelic psychotherapy. In: Winkelman MJ, Roberts TB editors. Psychedelic medicine: new evidence for hallucinogenic substances as treatments. Westport (CT): Praeger Publishers/Greenwood Publishing Group; 2007. p. 67–85.

[8] Goldfine CE, Tom JJ, Im DD, Yudkoff B, Anand A, Taylor JJ, Chai PR, Suzuki J. The therapeutic use and efficacy of ketamine in alcohol use disorder and alcohol withdrawal syndrome: a scoping review. Front Psych. 2023;14:1141836. doi:10.3389/fpsyt.2023.1141836. PMC10172666 37181899

[9] Orhurhu V, Orhurhu MS, Bhatia A, Cohen SP. Ketamine infusions for chronic pain: a systematic review and meta-analysis of randomized controlled trials. Anesth Analg. 2019;129(1):241–54. doi:10.1213/ANE.0000000000004185. 31082965

[10] Andrade C. Ketamine for depression, 4: in what dose, at what rate, by what route, for how long, and at what frequency?: (clinical and practical psychopharmacology). J Clin Psychiatry. 2017;78(7):e852–e857. doi:10.4088/JCP.17f11738. 28749092

[11] Doyle L, McCabe C, Keogh B, Brady A, McCann M. An overview of the qualitative descriptive design within nursing research. J Nurs Res. 2019;25(5):443–55. doi:10.1177/1744987119880234. PMC7932381 34394658

[12] DeJonckheere M, Vaughn LM. Semistructured interviewing in primary care research: a balance of relationship and rigour. Fam Med Comm Health. 2019;7(2):e000057. doi:10.1136/fmch-2018-000057. PMC6910737 32148704

[13] QSR International Pty Ltd. NVivo (Version 12). Comput Softw. Melbourne (AU): QSR International Pty Ltd; 2018.

[14] Elo S, Kyngäs H. The qualitative content analysis process. J Adv Nurs. 2008;62(1):107–15. doi:10.1111/j.1365-2648.2007.04569.x. 18352969

[15] Saunders B, Sim J, Kingstone T, Baker S, Waterfield J, Bartlam B, Burroughs H, Jinks C. Saturation in qualitative research: exploring its conceptualization and operationalization. Qual Quant. 2018;52(4):1893–907. doi:10.1007/s11135-017-0574-8. 29937585 PMC5993836

[16] Guest G, Bunce A, Johnson L. How many interviews are enough?: an experiment with data saturation and variability. Fld Meth. 2006;18:59–82. doi:10.1177/1525822X05279903.

[17] Shetty A, Delanerolle G, Cavalini H, Deng C, Yang X, Boyd A, Fernandez T, Phiri P, Bhaskar A, Shi JQ. A systematic review and network meta-analysis of pharmaceutical interventions used to manage chronic pain. Sci Rep. 2024;14(1):1621. doi:10.1038/s41598-023-49761-3. 38238384 PMC10796361

[18] Culp C, Kim HK, Abdi S. Ketamine use for cancer and chronic pain management. Front Pharmacol. 2021:11. doi:10.3389/fphar.2020.599721. PMC7941211 33708116

[19] Pergolizzi J, Ahlbeck K, Aldington D, Alon E, Coluzzi F, Dahan A, Huygen F, Kocot-Kępska M, Mangas AC, Mavrocordatos P, et al. The development of chronic pain: physiological CHANGE necessitates a multidisciplinary approach to treatment. Curr Med Res Opin. 2013;29(9):1127–35. doi:10.1185/03007995.2013.810615. 23786498 PMC3793283

[20] Luk KDK, Wan TWM, Wong YW, Cheung KMC, Chan KYK, Cheng ACS, Kwan MWW, Law KKP, Lee PWH, Cheing GLY. A multidisciplinary rehabilitation programme for patients with chronic low back pain: a prospective study. J Orthop Surg. 2010;18(2):131–38. doi:10.1177/230949901001800201. 20808000

[21] Tankha P. Ketamine for chronic pain: podcast transcript. Cleaveland Clinic. Cleaveland (Ohio): Cleaveland Clinic; 2024 2022 April 15 [Accessed 2024 November 15]. https://my.clevelandclinic.org/podcasts/neuro-pathways/ketamine-for-chronic-pain

[22] Cleaveland clinic: neurological institute outcomes. center for pain recovery ketamine infusions. cleaveland clinic. Cleaveland (Ohio): Cleaveland Clinic; 2023 [Accessed 2024 Nov 15]. https://my.clevelandclinic.org/departments/neurological/outcomes/1174-center-for-pain-recovery-ketamine-infusions

[23] Goel A, Kapoor B, Chan H, Ladha K, Katz J, Clarke H, Pazmino-Canizares J, Thomas Z, Philip K, Mattina G, et al. Psychotherapy for ketamine’s enhanced durability in chronic neuropathic pain: protocol for a pilot randomized controlled trial. JMIR Res Protoc. 2024;13:e54406. doi:10.2196/54406. 38630524 PMC11063874

[24] Duprat JA. Contributor: destigmatizing ketamine for legitimate pain use. AJMC. 2022. https://www.ajmc.com/view/contributor-destigmatizing-ketamine-for-legitimate-pain-use.

[25] Parikh SV, Vande Voort JL, Yocum AK, Achtyes E, Goes FS, Nykamp L, Singh B, Lopez-Vives D, Sera CE, Maixner D, et al. Clinical outcomes in the biomarkers of ketamine (Bio-K) study of open-label IV ketamine for refractory depression. J.Affect.Disord. 2024;348:143–51. doi:10.1016/j.jad.2023.12.033. 38142892

[26] Alnefeesi Y, Chen-Li D, Krane E, Jawad MY, Rodrigues NB, Ceban F, Di Vincenzo JD, Meshkat S, Rcm H, Gill H, et al. Real-world effectiveness of ketamine in treatment-resistant depression: a systematic review & meta-analysis. J Psychiatr Res. 2022;151:693–709. doi:10.1016/j.jpsychires.2022.04.037. 35688035

[27] Jilka S, Murray C, Wieczorek A, Griffiths H, Wykes T, McShane R. Exploring patients’ and carers’ views about the clinical use of ketamine to inform policy and practical decisions: mixed-methods study. BJPsych Open. 2019;5(5):e62. doi:10.1192/bjo.2019.52. 31530293 PMC6669880

[28] Jilka S, Odoi CM, Wilson E, Meran S, Simblett S, Wykes T. Ketamine treatment for depression: qualitative study exploring patient views. BJPsych Open. 2021;7(1):e32. doi:10.1192/bjo.2020.165. 33427156 PMC8058884

[29] Ead H. Low-dose ketamine: overcoming stigmas to optimize pain management. Canadian Nurse. Ottawa (Ontario): Canadian Nurses Association; 2024 2022 September 6 [Accessed 2024 November 20]. https://www.canadian-nurse.com/blogs/cn-content/2022/09/06/low-dose-ketamine-overcoming-stigmas

[30] Pittera B Ketamine and treatment resistant depression: researching psychologists’ perceptions regarding the use of ketamine for treatment resistant depression [dissertation]. Los Angeles (USA): Alliant International University; 2024. https://www.proquest.com/docview/3046408077?sourcetype=Dissertations%20&%20Theses

[31] Stockwell G Understanding the experience of ketamine-assisted therapy and the importance of context: a qualitative study [thesis]. Auckland (Australia): The University of Auckland; 2023. https://researchspace.auckland.ac.nz/server/api/core/bitstreams/69239273-5f9a-47d3-8c32-9fa1ca8ce432/content

[32] Breeksema JJ, Niemeijer A, Kuin B, Veraart J, Kamphuis J, Schimmel N, Van Den Brink W, Vermetten E, Schoevers RA. Holding on or letting go? Patient experiences of control, context, and care in oral esketamine treatment for treatment-resistant depression: a qualitative study. Front Psychiatry. 2022:13. doi:10.3389/fpsyt.2022.948115. PMC9732097 36506427

[33] Frantsve LME, Kerns RD. Patient-provider interactions in the management of chronic pain: current findings within the context of shared medical decision making. Pain Med. 2007;8(1):25–35. doi:10.1111/j.1526-4637.2007.00250.x. 17244101

[34] Government of Canada. Best Brains Exchange report: treatment of chronic pain and complex concurrent mental health and substance use conditions. Ottawa; 2023 September. [accessed 2024 Nov 10]. https://www.canada.ca/en/health-canada/services/publications/healthy-living/best-brains-exchange-report-2023.html#a3.2

[35] Hoffman PK, Meier BP, Council JR. A comparison of chronic pain between an urban and rural population. J Community Health Nurs. 2002;19(4):213–24. doi:10.1207/S15327655JCHN1904_02. 12494742

[36] Bath B, Trask C, McCrosky J, Lawson J. A biopsychosocial profile of adult Canadians with and without chronic back disorders: a population-based analysis of the 2009-2010 Canadian Community Health Surveys. BioMed Res Int. 2014;2014:1–11. doi:10.1155/2014/919621. PMC4058275 24971357

[37] Yin Z, Li S, Ortega C, Bobadilla R, Winkler PL, Hernández AE, Simmonds MJ. Impacts on patient-centered outcomes of a chronic pain self-management program in a rural community: a feasibility study. Geriatr Nurs. 2021;42(5):1198–203. doi:10.1016/j.gerinurse.2021.06.026. 34425422

[38] Ketamine Mobile. Mobile ketamine treatments. California (USA); 2024 [Accessed 2024 Nov 20]. https://ketaminemobile.com/service/#.

[39] National Guideline Centre (UK). Evidence review for communication between healthcare professionals and people with chronic pain (chronic primary pain and chronic secondary pain): chronic pain (primary and secondary) in over 16s: assessment of all chronic pain and management of chronic primary pain. London: National Institute for Health and Care Excellence (NICE); 2021. 33939360

[40] Henry SG, Matthias MS. Patient-clinician communication about pain: a conceptual model and narrative review. Pain Med. 2018;19(11):2154–65. doi:10.1093/pm/pny003. 29401356 PMC6454797

